# The Association of Preoperative Patient Resilience With Postoperative Patient-Reported Outcomes Following Total Joint Arthroplasty: A Systematic Review and Meta-Analysis

**DOI:** 10.7759/cureus.110469

**Published:** 2026-06-08

**Authors:** Shaheryar Asad, Jean Shanaa, Guneet S Bindra, Sarah Lateef, Leeann Qubain, J. Brock Walker

**Affiliations:** 1 Orthopedic Surgery, California Northstate University College of Medicine, Elk Grove, USA; 2 Neurology, California Northstate University College of Medicine, Elk Grove, USA; 3 Ophthalmology, New York Institute of Technology College of Osteopathic Medicine, Jonesboro, USA; 4 Orthopedic Surgery, The University of Arizona College of Medicine - Phoenix, Phoenix, USA

**Keywords:** arthroplasty, resilience, tha, tja, tka

## Abstract

Total joint arthroplasty (TJA) for advanced joint disease is associated with a substantial recovery period. Psychological resilience, defined as the capacity to adapt positively to adversity, may play an important role in shaping patient perceptions and postoperative outcomes. In this systematic review and meta-analysis, we hypothesized that higher preoperative resilience would be associated with better patient-reported outcome measures (PROMs) following TJA.

A comprehensive search of the databases PubMed, Embase, and Scopus identified 594 studies, of which nine met the inclusion criteria and examined the relationship between preoperative resilience and postoperative outcomes in TJA patients. These studies, published between 2019 and 2022, included a total of 1,328 patients with a pooled mean age of 67.5 years. Resilience was assessed using the Brief Resilience Scale (BRS) in six studies and the Connor-Davidson Resilience Scale (CD-RISC) in three studies. Assessment of methodological quality demonstrated a low to moderate risk of bias across the included studies.

Higher preoperative resilience was significantly associated with better outcomes across multiple measures, including the Patient-Reported Outcomes Measurement Information System (PROMIS) Physical Health (PH) and Mental Health (MH) (both p < 0.001), the Knee Injury and Osteoarthritis Outcome Score (KOOS) (p < 0.05), the EuroQol Group 5 Dimension (EQ-5D) (p < 0.001), the EuroQol Group Visual Analogue Scale (EQ-VAS) (p < 0.001), and the Western Ontario and McMaster Universities Osteoarthritis Index (WOMAC) (p < 0.05). Meta-analysis further confirmed significant associations between resilience and PROMIS-PH, PROMIS-MH, and KOOS scores.

Overall, greater preoperative resilience is associated with better postoperative PROMs following TJA. These findings suggest that resilience may be a potentially modifiable factor and highlight the value of a more holistic preoperative approach. However, variability in measurement tools across studies underscores the need for standardized assessment methods. Future research should aim to clarify the mechanisms by which resilience influences recovery and to develop targeted interventions to enhance preoperative resilience and optimize surgical outcomes.

## Introduction and background

Total joint arthroplasty (TJA), which includes total knee arthroplasty (TKA) and total hip arthroplasty (THA) in the present study, is an effective intervention for reducing pain and improving function in patients with severe joint disease. These procedures are becoming increasingly prevalent, with THAs projected to increase by 659% and TKAs projected to increase by 469% by 2060 [1]. Given this rapid rise in utilization, optimizing postoperative recovery and long-term outcomes has become a critical priority for both patients and healthcare systems.

Despite its widespread use and benefits, TJA is associated with considerable clinical and economic impacts. In the United States, the average cost of a primary TKA ranged from $17,000 to $30,000 in 2014 [2]. The procedure itself, postoperative care, and rehabilitation all contribute to the overall expenditure. Furthermore, complications may include infection, prosthesis-related issues, and thromboembolic events. Limiting recovery time is therefore essential, as it is associated with a lower risk of complications, improved mental well-being, and enhanced quality of life [3]. Importantly, recovery is not solely determined by surgical and medical factors; patient engagement with rehabilitation, adherence to postoperative protocols, and psychological readiness also play key roles in influencing outcomes.

Psychological factors have been increasingly recognized as important determinants of postoperative recovery; however, they are not routinely incorporated into perioperative planning [4]. Among these, resilience represents a distinct construct. Unlike related psychological factors such as anxiety, depression, or pain catastrophizing, which primarily reflect negative emotional states or maladaptive thought patterns, resilience is defined as the capacity to adapt positively to stress or adversity. It reflects an individual's ability to cope, recover, and maintain psychological stability despite challenges. Clinically, higher resilience may translate into better engagement with rehabilitation, improved coping with postoperative pain, and greater adherence to recovery protocols.

In this review, we focus on the role of preoperative resilience and its impact on patient-reported outcome measures (PROMs) following TJA. PROMs are standardized tools used to assess outcomes from the patient's perspective, capturing domains such as pain, physical function, and quality of life. Commonly used instruments in TJA include the Patient-Reported Outcomes Measurement Information System (PROMIS) Physical Health (PH) and Mental Health (MH) [5,6,7], the Western Ontario and McMaster Universities Osteoarthritis Index (WOMAC) [8,9], and the Knee Injury and Osteoarthritis Outcome Score (KOOS) [10,5,6]. These measures are critical for evaluating surgical success beyond traditional clinical metrics.

Preoperative patient resilience may influence various aspects of the surgical experience, including psychological preparedness, coping mechanisms, and recovery trajectories [11]. Moreover, increased resilience has been correlated with more favorable PROMs, including reduced pain, improved physical function, and better quality of life in other orthopedic populations, such as patients with elbow fractures [12].

Unfortunately, while numerous preoperative factors are known to contribute to postoperative outcomes, there remains a paucity of literature specifically examining the role of preoperative resilience in TJA. Although a recent study by Kim et al. investigated the impact of preoperative resilience on postoperative outcomes after TJA, the existing literature has not yet been quantitatively synthesized in a meta-analysis. Additionally, while Kim et al. evaluated opioid usage, we limited our analysis to PROMs to reduce heterogeneity, as resilience may influence PROMs and opioid use through distinct mechanisms [13]. Given these considerations, we hypothesize that higher levels of preoperative resilience in patients undergoing TJA are associated with significantly better PROMs.

## Review

Methods

Study Design and Registration

This systematic review was conducted in accordance with the Preferred Reporting Items for Systematic Reviews and Meta-Analyses (PRISMA) guidelines and was prospectively registered with PROSPERO (ID: CRD42024555782).

Information Sources and Search Strategy

A systematic literature search was performed on PubMed, Embase, and Scopus from database inception to January 2025 using the Boolean terms: "resilience AND (Total Joint Arthroplasty OR (knee OR Total Knee Arthroplasty OR TKA) OR (hip OR Total Hip Arthroplasty OR THA))[14]." Two reviewers (SA and JS) independently screened titles and abstracts, followed by a full-text review to identify studies evaluating preoperative resilience and its association with PROMs in patients undergoing TJA. Eligible studies were limited to peer-reviewed articles reporting primary data and published in English, while case reports, case series, reviews, abstracts, editorials, posters, and letters were excluded [15]. Reference lists of included studies were manually screened to identify additional relevant articles. Further details regarding the specific search terms used, the PICOS criteria, and the inclusion/exclusion criteria are provided in Tables 1-3.

**Table 1:** Database-specific search strategy

Database	Search strategy	Search period
PubMed	("Resilience, Psychological"[MeSH] OR resilience[tiab]) AND ("Arthroplasty, Replacement"[MeSH] OR "Arthroplasty, Replacement, Knee"[MeSH] OR "Arthroplasty, Replacement, Hip"[MeSH] OR "total joint arthroplasty"[tiab] OR "total knee arthroplasty"[tiab] OR TKA[tiab] OR "total hip arthroplasty"[tiab] OR THA[tiab] OR knee[tiab] OR hip[tiab])	Database inception to January 2025
Embase	('psychological resilience'/exp OR resilience:ti,ab) AND ('arthroplasty'/exp OR 'knee arthroplasty'/exp OR 'hip arthroplasty'/exp OR 'total joint arthroplasty':ti,ab OR 'total knee arthroplasty':ti,ab OR tka:ti,ab OR 'total hip arthroplasty':ti,ab OR tha:ti,ab OR knee:ti,ab OR hip:ti,ab)	Database inception to January 2025
Scopus	TITLE-ABS-KEY (resilience) AND TITLE-ABS-KEY ("total joint arthroplasty" OR "total knee arthroplasty" OR TKA OR "total hip arthroplasty" OR THA OR knee OR hip)	Database inception to January 2025

**Table 2:** PICOS criteria PICOS: Population, Intervention, Comparison, Outcomes and Study

Component	Description
Population	Patients undergoing total joint arthroplasty (hip or knee)
Intervention/exposure	Preoperative psychological resilience
Comparison	Lower resilience vs. higher resilience
Outcomes	Patient-reported outcomes (PROMs), pain, functional outcomes, complications
Study design	Observational studies, clinical studies, and randomized controlled trials

**Table 3:** Inclusion and exclusion criteria

Inclusion criteria	Exclusion criteria
Studies evaluating resilience in total joint arthroplasty (hip or knee) patients	Case reports, reviews, editorials
Studies reporting clinical or patient-reported outcome measures	Non-English studies
Human subjects	Animal studies
Peer-reviewed articles	Conference abstracts without full text
Primary research studies	Studies not involving hip or knee arthroplasty

Eligibility Criteria

Eligibility criteria were predefined using the PICOS framework and are detailed in Tables 2-3.

Selection Process

Study selection was performed by two independent reviewers (SA and JS), with disagreements resolved through discussion and consensus.

Data Collection Process and Data Items

Data were extracted from all included studies. Collected variables included patient demographics (age, sex), type of arthroplasty procedure, measures of preoperative resilience, and reported PROMs. Primary outcomes included preoperative and/or postoperative scores from WOMAC [8,9], UCLA [8,9], Knowledge [8,9], HOOS-JR [5], KOOS/KOOS-JR [10,5,6], PROMIS-PH and PROMIS-MH [5,6,7], EuroQol Group 5 Dimension (EQ-5D) [5], and EuroQol Group Visual Analogue Scale (EQ-VAS) [5]. The secondary outcome was length of stay (LOS) or time to discharge.

Risk of Bias Assessment

Methodological quality and risk of bias were evaluated using the Methodological Index for Non-Randomized Studies (MINORS). Each item is scored as 0 (not reported), 1 (reported but inadequate), or 2 (reported and adequate), with maximum scores of 16 for noncomparative studies and 24 for comparative studies. For noncomparative studies, scores < 8, 8-12, and 12-16 were classified as high, moderate, and low risk of bias, respectively [15]. For comparative studies, scores < 16, 16-20, and 21-24 were classified as high, moderate, and low risk of bias, respectively [16].

Certainty of Evidence

The overall certainty of evidence for each outcome was assessed using the Grading of Recommendations Assessment, Development and Evaluation (GRADE) framework.

Effect Measures and Synthesis Methods

Descriptive statistics, including means, standard deviations (SDs), ranges, medians, 95% confidence intervals (CIs), and correlation coefficients, were reported when available. For quantitative synthesis, the primary effect size was the Pearson correlation coefficient (r) describing the association between preoperative resilience and postoperative PROMs.

When studies reported correlation coefficients, these were transformed using Fisher’s z transformation before pooling to stabilize variance, and pooled estimates were subsequently back-transformed to r for interpretation. For studies reporting group-based comparisons (e.g., high vs. low resilience), effect sizes were converted to standardized mean differences (SMDs; Hedges g) when sufficient data were available. For studies reporting pre- and postoperative changes, mean change scores were used when reported, or estimated using available summary statistics. When necessary, effect sizes were derived from reported statistics, including CIs, standard errors, or p-values, using established methods.

Meta-analyses were performed using a random-effects model (DerSimonian and Laird method) to account for between-study heterogeneity and were conducted only when at least two studies reported comparable outcomes. A random-effects model was selected to account for anticipated clinical and methodological heterogeneity across studies, including differences in patient populations, resilience measurement tools, and outcome measures. The DerSimonian and Laird method was used due to its widespread application in meta-analyses of observational studies and its ability to incorporate between-study variability.

Heterogeneity was assessed using the I² statistic, which quantifies the proportion of total variation attributable to between-study differences rather than chance. Correlation coefficients were transformed using Fisher's z transformation to stabilize variance and improve the accuracy of pooled estimates, particularly when combining studies with varying sample sizes. Statistical heterogeneity was assessed using the I² statistic [16]. Publication bias was not formally assessed using funnel plots due to the limited number of studies included in each meta-analysis (fewer than 10). Forest plots were generated using R (version 4.3.1; R Foundation for Statistical Computing, Vienna, Austria) and RStudio (Posit Software, Boston, MA), and statistical significance was defined as a two-sided p-value < 0.05 [15].

Results

Search Results, Study Characteristics, and Demographic Data

**Figure 1:**
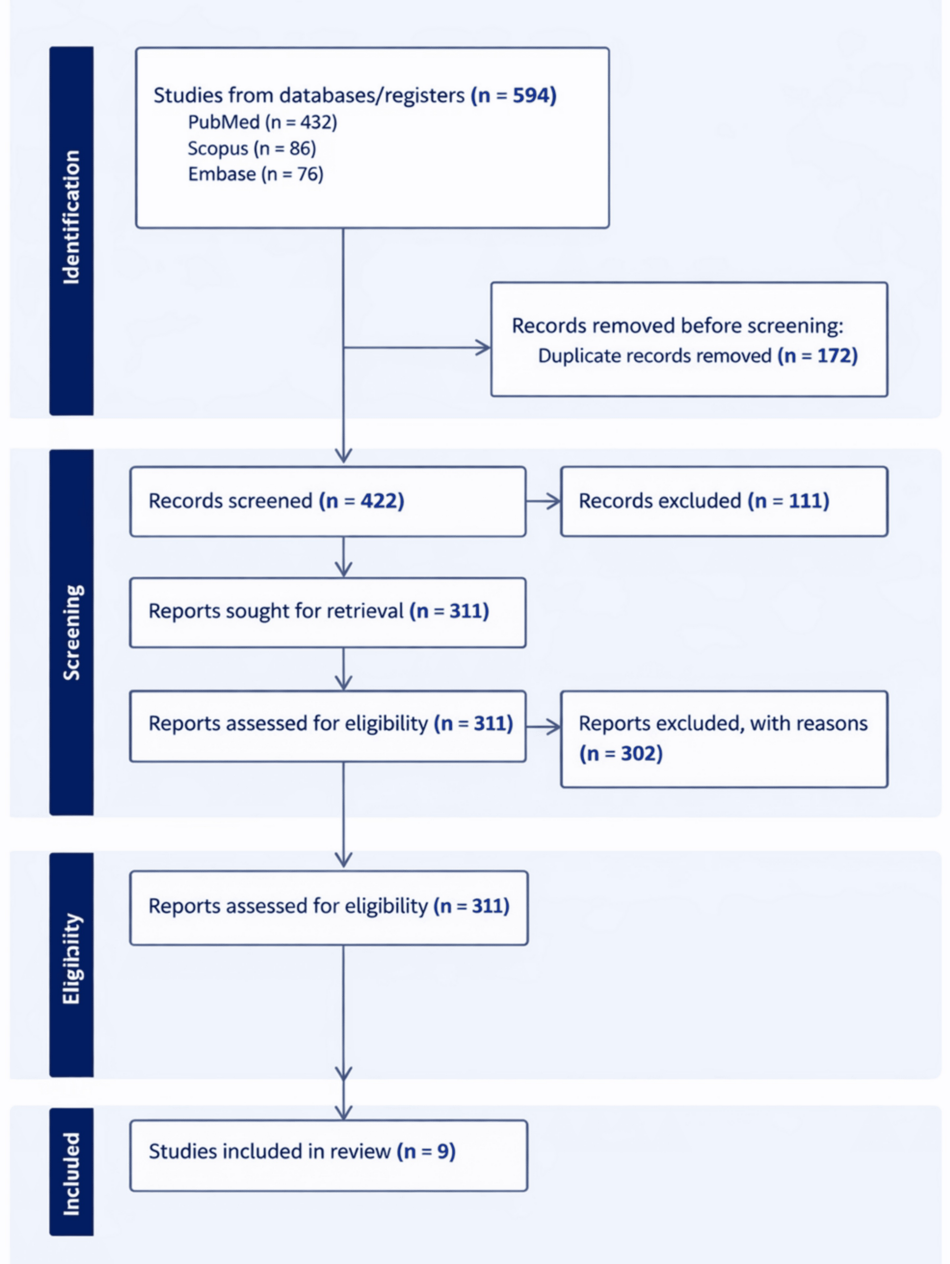
PRISMA flow diagram depicting the article selection process PRISMA: Preferred Reporting Items for Systematic Reviews and Meta-Analyses

The initial search yielded 594 articles. After the removal of duplicates, 422 unique studies remained, of which nine met the inclusion criteria (Figure 1). All included studies were published between 2019 and 2022, comprising a total of 1,328 patients. Eight studies reported sex distribution, including 520 males (44.6%) and 645 females (55.4%).

Preoperative resilience was assessed using the Brief Resilience Scale (BRS) [10,5,6,7,17,5] in six studies and the Connor-Davidson Resilience Scale (CD-RISC) [8,9,18] in three studies. Eight studies reported patient age, with a pooled mean of 67.5 years (range: 66.2-68.8 years) [14]. Additionally, eight studies included follow-up data. Detailed methodological quality, study characteristics, and demographic information are summarized in Tables 4-5.

**Table 4:** Characteristics of the included studies

Study title	Author	Year	Journal	Study design	Risk of Bias
Higher patient knowledge and resilience improve the functional outcome of primary total knee arthroplasty	Bumberger et al. [11]	2021	Wiener klinische Wochenschrift-The Central European Journal of Medicine	Retrospective review	Low
Specific knowledge and resilience affect short-term outcome in patients following primary total hip arthroplasty	Bumberger et al. [12]	2022	Archives of Orthopaedic and Trauma Surgery	Cross-sectional prospective study	Moderate
Patient resilience has moderate correlation with functional outcomes, but not satisfaction, after primary unilateral total knee arthroplasty	Haffar et al. [13]	2021	Arthroplasty Today	Prospective longitudinal study	Moderate
The influence of patient resilience and health status on satisfaction after total hip and knee arthroplasty	Lynskey et al. [14]	2021	Surgeon	Prospective cohort study	Moderate
Impact of resilience on outcomes of total knee arthroplasty	Magaldi et al. [15]	2019	The Journal of Arthroplasty	Prospective cohort study	Moderate
Does resilience predict hospital length of stay after total knee arthroplasty? A prospective observational cohort study	March et al. [16]	2022	Arthroplasty	Prospective observational cohort study	Moderate
Resilience and pain catastrophizing among patients with total knee arthroplasty: a cohort study to examine psychological constructs as predictors of post-operative outcomes	Nwanko et al. [17]	2021	Health and Quality of Life Outcomes	Prospective cohort study	Moderate
Resilience and depression influence clinical outcomes following primary total joint arthroplasty	Trinh et al. [18]	2021	The Journal of Arthroplasty	Prospective cohort study	Moderate
The impact of patient resilience on discharge after total hip arthroplasty	Zabat et al. [19]	2022	The Journal of Arthroplasty	Retrospective review	Moderate

**Table 5:** Methodological assessment, study characteristics, and demographic data SD: standard deviation; MINORS: Methodological Index for Non-Randomized Studies; CD-RISC: Connor-Davidson Resilience Scale [8,9,18]; WOMAC: Western Ontario and McMaster Universities Osteoarthritis Index [8,9]; BRS: Brief Resilience Scale [10,5,6,7,19,17]; DASS-21: Depression Anxiety Stress Scale-21 [19]

Study	Authors and Year	Total number of patients	Average age (range) or ± SD, years	Sex demographics (female, n; %)	Methodology for resilience measurement	Length of follow-up (postoperative)	MINORS scores
Higher patient knowledge and resilience improve the functional outcome of primary total knee arthroplasty	Bumberger et al. (2021) [11]	163	70 (40-87)	Unknown	CD-RISC, WOMAC, UCLA	8 weeks	14
Specific knowledge and resilience affect short-term outcome in patients following primary total hip arthroplasty	Bumberger et al. (2022) [12]	103	67.5 (38-88)	54; 52.4%	CD-RISC	2 months, 6 months, 12 months	16
Patient resilience has moderate correlation with functional outcomes, but not satisfaction, after primary unilateral total knee arthroplasty	Haffar et al. (2021) [13]	86	66 (58.5-71.0)	37; 43.0%	BRS	1-2 years	13
The influence of patient resilience and health status on satisfaction after total hip and knee arthroplasty	Lynskey et al. (2021) [14]	140	69 (44-91)	84; 60%	CD-RISC	3 months, 1-5 years	10
Impact of resilience on outcomes of total knee arthroplasty	Magaldi et al. (2019) [15]	153	68.2 ± 8.3	79; 51.6%	BRS	3 months, 12 months	12
Does resilience predict hospital length of stay after total knee arthroplasty? A prospective observational cohort study	March et al. (2022) [16]	75	68 ± 8.2	49; 65.3%	BRS, DASS-21	N/A	15
Resilience and pain catastrophizing among patients with total knee arthroplasty: a cohort study to examine psychological constructs as predictors of post-operative outcomes	Nwanko et al. (2021) [17]	117	67 (35-85)	62; 53%	BRS	3 months	15
Resilience and depression influence clinical outcomes following primary total joint arthroplasty	Trinh et al. (2021) [18]	98	64 (54-69)	55, 56.1%	BRS	1 year	11
The impact of patient resilience on discharge after total hip arthroplasty	Zabat et al. (2022) [19]	398 (low resilience: 28; normal resilience: 274; high resilience: 91)	Low resilience: 66.50 ± 10.63; normal resilience: 63.55 ± 12.24; high resilience: 62.32 ± 10.48	225; 57.3% (low resilience: 20, 71.4%; normal resilience: 161, 58.7%; high resilience: 44, 48.4%)	BRS	3 months	11

Methodological Assessment, Risk of Bias, and Grade Analysis

The mean MINORS score for the nine studies was 13 ± 2.12 (10.88 - 15.12). One study was deemed to have a low risk of bias, whereas eight studies were deemed to have a moderate risk of bias. No studies had a high risk of bias. The certainty of evidence for each outcome was assessed using the GRADE framework. The overall certainty of evidence, as assessed using the GRADE framework, ranged from low to very low across outcomes. This was primarily driven by the observational design of included studies, moderate risk of bias, imprecision due to limited sample sizes, and variable heterogeneity across pooled analyses. A detailed assessment of the MINORS score and GRADE analysis is presented in Tables 6-7.

**Table 6:** MINORS criteria and bias assessment MINORS: Methodological Index for Non-Randomized Studies

Author	Year published	Title	Criteria 1	Criteria 2	Criteria 3	Criteria 4	Criteria 5	Criteria 6	Criteria 7	Criteria 8	Criteria 9	Criteria 10	Criteria 11	Criteria 12	Total	Risk of bias
Trinh et al. [18]	2021	Resilience and depression influence clinical outcomes following primary total joint arthroplasty	2	2	2	2	0	1	0	0	N/A	N/A	N/A	2	11	Moderate
Magaldi et al. [15]	2019	Impact of resilience on outcomes of total knee arthroplasty	2	2	2	2	0	2	0	0	0	N/A	N/A	2	12	Moderate
Lynskey et al. [14]	2021	The influence of patient resilience and health status on satisfaction after total hip and knee arthroplasty	2	1	1	2	0	2	0	0	0	N/A	N/A	2	10	Moderate
Nwanko et al. [17]	2021	Resilience and pain catastrophizing among patients with total knee arthroplasty: a cohort study to examine psychological constructs as predictors of post-operative outcomes	2	2	2	2	0	2	1	2	0	0	0	2	15	Moderate
March et al. [16]	2022	Does resilience predict hospital length of stay after total knee arthroplasty? A prospective observational cohort study	2	2	2	2	0	2	1	2	0	0	0	2	15	Moderate
Bumberger et al. [12]	2022	Specific knowledge and resilience affect short-term outcome in patients following primary total hip arthroplasty	2	2	2	2	0	2	2	2	0	0	0	2	16	Moderate
Zabat et al.	2022	The impact of patient resilience on discharge after total hip arthroplasty	2	2	0	2	0	2	1	0	0	0	0	2	11	Moderate
Bumberger et al. [11]	2021	Higher patient knowledge and resilience improve the functional outcome of primary total knee arthroplasty	2	2	2	2	2	1	1	0	0	0	0	2	14	Low
Haffar et al. [13]	2021	Patient resilience has moderate correlation with functional outcomes, but not satisfaction, after primary unilateral total knee arthroplasty	2	2	0	2	0	2	1	2	0	0	0	2	13	Moderate

**Table 7:** GRADE analysis GRADE: Grading of Recommendations Assessment, Development and Evaluation; PROMIS-PH: Patient-Reported Outcomes Measurement Information System-Physical Health; PROMIS-MH: Patient-Reported Outcomes Measurement Information System-Mental Health; KOOS: Knee Injury and Osteoarthritis Outcome Score

Outcome	No. of studies	Study design	Risk of bias	Inconsistency	Indirectness	Imprecision	Publication bias	Overall certainty
Preoperative PROMIS-PH	2	Observational	Serious	Not serious (I² = 0%)	Not serious	Serious	Undetected	Low
Preoperative PROMIS-MH	2	Observational	Serious	Moderate (I² = 55%)	Not serious	Serious	Undetected	Very low
3-month KOOS	2	Observational	Serious	Moderate (I² = 60%)	Not serious	Serious	Undetected	Very low
3-month PROMIS-PH	2	Observational	Serious	Moderate (I² = 49%)	Not serious	Serious	Undetected	Very low
3-month PROMIS-MH	2	Observational	Serious	Moderate (I² = 44%)	Not serious	Serious	Undetected	Very low
12-month PROMIS-PH	2	Observational	Serious	Moderate (I² = 62%)	Not serious	Serious	Undetected	Very low
12-month PROMIS-MH	2	Observational	Serious	Not serious (I² = 0%)	Not serious	Serious	Undetected	Low

Patient-Reported Outcomes at Two to Three Months

The study by Trinh et al. found that higher preoperative BRS scores in patients undergoing TJA are significantly correlated with higher preoperative PROMIS-PH and PROMIS-MH (r = +0.38, p < 0.001, r = +0.50, p < 0.001, respectively) scores [7]. Lynskey et al. found that patients undergoing THA or TKA with a higher resilience had a significant correlation with improvement in their health status (r = +0.530; p < 0.001) [18]. The study by Bumberger et al. in 2022 reported that higher knowledge and resilience scores were associated with improved WOMAC scores at two months after THA. This study also found a significant correlation between WOMAC scores and knowledge and resilience (r = -0.231, p = 0.012; r = -0.248, p = 0.008, respectively), and between UCLA scores and knowledge (r = 0.279, p = 0.003) [9].

Magaldi et al. found a significant change in KOOS-JR, PROMIS-PH, and EQ-5D scores preoperatively compared to postoperative scores obtained at three months (p < 0.001, p < 0.001, p < 0.001, respectively) for patients undergoing TKA. The authors also assessed preoperative resilience levels using the BRS scale and assessed the variance of preoperative resilience with postoperative outcomes at 3 months (KOOS-JR: R2 = 0.016, p = 0.098; PROMIS-PH: R2 = 0.061, p < 0.001; PROMIS-MH: R2 = 0.145, p < 0.001; EQ-5D: R2 = 0.100, p < 0.001) [5].

This was corroborated by the findings from Nwankwo et al., in which they reported significant Pearson correlation coefficients for BRS with KOOS (r = 0.29, 95% CI: 0.11-0.47, p = 0.002), PROMIS-PH (r = 0.32, 95% CI: 0.21 - 0.54, p < 0.001), and PROMIS-MH (r = 0.64, 95% CI: 0.33 - 0.63, p < 0.001) preoperatively in patients undergoing TKA. The study also reported significant unadjusted Pearson correlation coefficients for BRS with knee function measured by KOOS (r = 0.31, 95% CI: 0.11 - 0.50, p = 0.002), physical health measured by PROMIS-PH (r = 0.40, 95% CI: 0.21 - 0.58, p < 0.001), and mental health measured by PROMIS-MH (r = 0.51, 95% CI: 0.33 - 0.68, p < 0.001) at the three-month follow-up visit [6]. Furthermore, Zabat et al. reported HOOS-JR scores that improved significantly at the three-month follow-up. With patients stratified into groups based on their level of resilience, the scores improved by 4.7% and 11.7% (p = 0.03) for normal and high resilience patients, respectively (low resilience: 72.5 ± 16.3; normal resilience: 75.9 ± 15.5; high resilience: 81.0 ± 12.7) [17].

**Figure 2:**
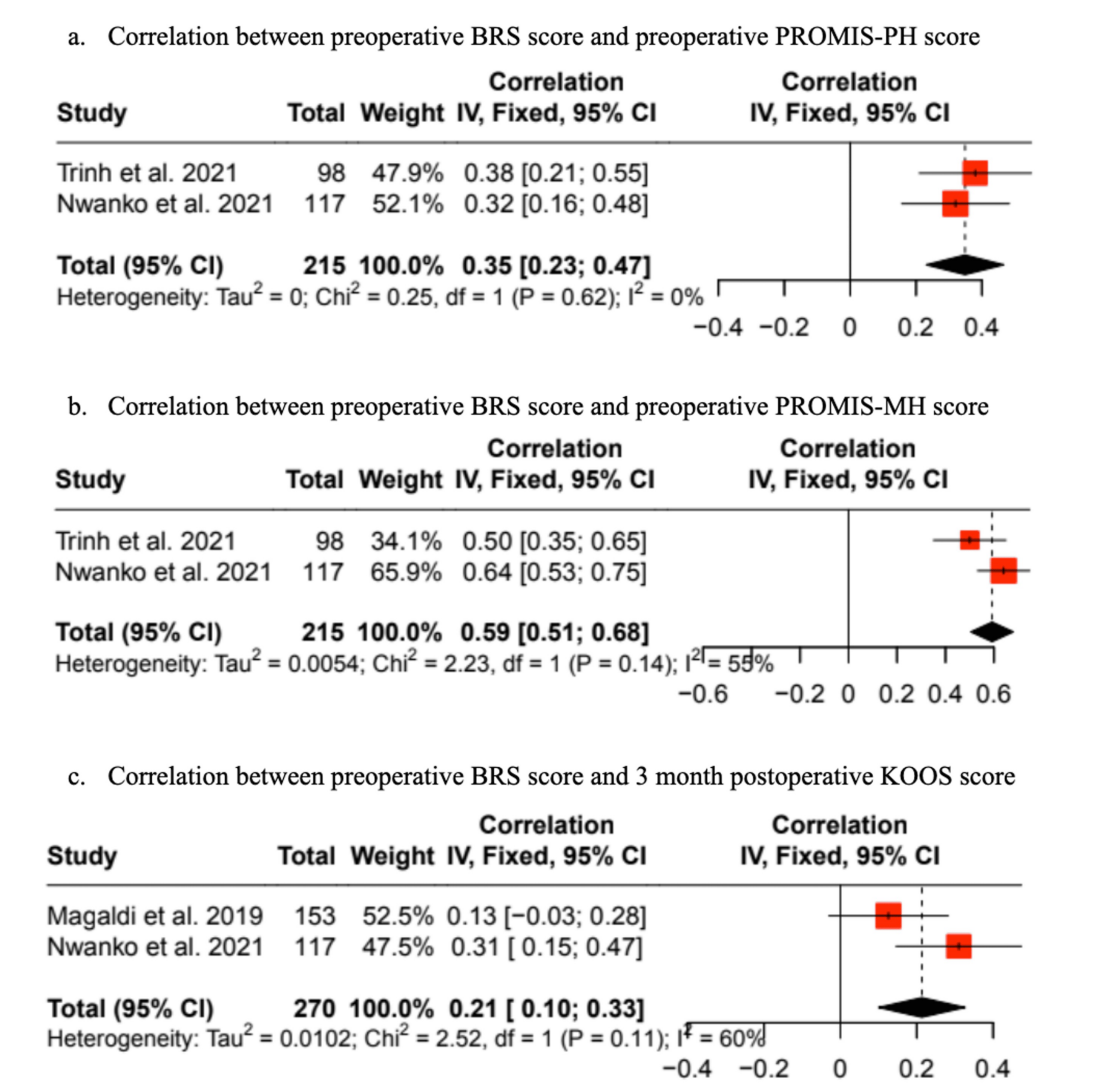
Forest plot for correlation - 1 Forest plot for the correlation between (a) preoperative BRS score and preoperative PROMIS-PH score, (b) preoperative BRS score and preoperative PROMIS-MH score, and (c) preoperative BRS score and 3-month postoperative KOOS score. Data were derived from Trinh et al. [7], Nwankwo et al. [6], and Magaldi et al. [5]. BRS: Brief Resilience Scale; PROMIS-PH: Patient-Reported Outcomes Measurement Information System-Physical Health; PROMIS-MH: Patient-Reported Outcomes Measurement Information System-Mental Health; KOOS: Knee Injury and Osteoarthritis Outcome Score; CI: confidence interval

**Figure 3:**
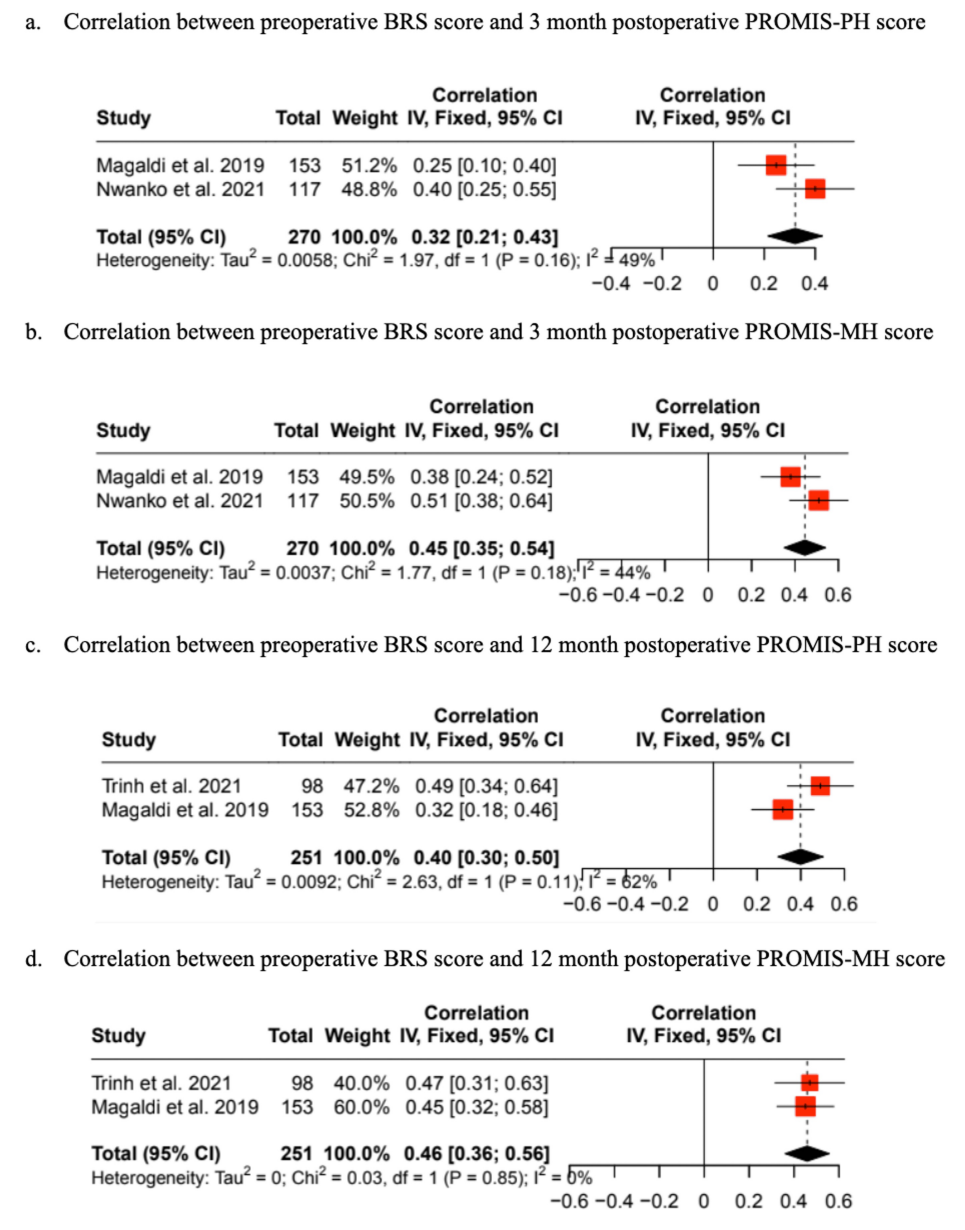
Forest plot for correlation - 2 Forest plot for the correlation between (a) preoperative BRS score and 3-month postoperative PROMIS-PH score, (b) preoperative BRS score and 3-month postoperative PROMIS-MH score, (c) preoperative BRS score and 12-month postoperative PROMIS-PH score, and (d) preoperative BRS score and 12-month postoperative PROMIS-MH score. Data were derived from Trinh et al. [7], Nwankwo et al. [6], and Magaldi et al. [5]. BRS: Brief Resilience Scale; PROMIS-PH: Patient-Reported Outcomes Measurement Information System-Physical Health; PROMIS-MH: Patient-Reported Outcomes Measurement Information System-Mental Health; CI: confidence interval

In the meta-analysis of the correlation data in the studies by Trinh et al. and Nwankwo et al., a significant correlation between preoperative BRS score and preoperative PROMIS-PH and PROMIS-MH scores was observed [7,6]. Another meta-analysis of the correlation data from Magaldi et al. and Nwankwo et al. demonstrated a significant correlation between preoperative BRS score and three-month postoperative KOOS, PROMIS-PH, and PROMIS-MH scores (Figures 2, 3) [5,6].

Contributions of Resilience to Longer-Term Outcomes

In their 2021 study, Bumberger et al. found that UCLA scores on admission for rehabilitation were significantly predicted by knowledge and resilience scores (p = 0.013). WOMAC scores were reported to have significant variance with knowledge and age (R2 = 14.3%, p = 0.003), whereas UCLA scores were reported to have significant variance with knowledge and resilience (R2 = 13.8%, p = 0.013) [8]. The study by Magaldi et al. reported significant unadjusted Pearson correlation coefficients for BRS with various measures of knee function at 12 months (KOOS-JR: R2 = 0.018, p < 0.095; PROMIS-PH: R2 = 0.101, p < 0.001; PROMIS-MH: R2 = 0.204, p < 0.001; EQ-5D: R2 = 0.160, p < 0.001) follow-up visits. PROMIS-PH, PROMIS-MH, and EQ-5D all yielded significant correlations between preoperative resilience and follow-up visit scores [5]. Likewise, Trinh et al. found statistical significance between preoperative BRS and PROMIS-PH and PROMIS-MH scores at the one-year postoperative follow-up visit (r = +0.49, p < 0.001, r = +0.47, p < 0.001, respectively) [7].

Albeit using different methods, Haffar et al. demonstrated similar findings of a significant difference between preoperative and one-year postoperative self-reported outcomes. The authors determined preoperative values for the Veterans-Rand 12-Item Survey (VR-12 MCS) (58.5; range: 53.0 - 62.6), KOOS-JR (50.0; range: 42.3 - 58.8), Knee Society Scores (KSS) functional activities (43.7 ± 15.2), KSS patient satisfaction (14.0; range: 10.0 - 18.0), KSS patient expectations (15.0; range: 14.0 - 15.0), and KSS Symptom Scores (8.24; range: 4.11 - 11.6) for patients undergoing unilateral TKA. The study also reported the one-year postoperative values for VR-12 MCS (62.1; range: 57.4 - 63.7), KOOS-JR (79.9; range: 70.7 - 92.0), KSS functional activities (81.0; range: 72.5 - 90.0), KSS patient satisfaction (36.0; range: 30.0 - 40.0), KSS patient expectations (11.0; range: 9.0 - 13.0), KSS symptom scores (22.0; range: 20.0 - 24.0). The study found a significant difference (p < 0.008) for VR-12 MCS scores and for KOOS-JR, KSS functional activities, KSS patient satisfaction, KSS patient expectations, and KSS symptom scores (p < 0.001) between preoperative and one-year postoperative values. The study also reported a significant correlation between resilience measured by BRS and preoperative VR-12 MCS (r = 0.270, p = 0.013) and between resilience and postoperative VR-12 MCS (r = 0.428, p < 0.001) [10].

These noteworthy changes in outcomes were also shown during comparisons between patient groups with varying levels of resilience. Trinh et al. saw significant differences in PROMIS-MH scores among low, normal, and high resilience groups preoperatively (43.5 vs. 48.3 vs. 53.3, p < 0.001, respectively) and at the one-year postoperative follow-up visit (45.8 vs. 50.8 vs. 53.3, p < 0.001, respectively). A significant difference in PROMIS-PH scores among low, normal, and high resilience groups was seen preoperatively (37.4 vs. 39.8 vs. 44.9, p = 0.002, respectively) and at one year postoperatively (42.3 vs. 47.7 vs. 54.1, p < 0.001, respectively) [7]. Table 3 provides a complete summary of the outcomes reported for each study.

Our meta-analysis of the correlation data from Trinh et al. and Magaldi et al. demonstrated a significant correlation between preoperative BRS scores and 12-month postoperative PROMIS-PH and PROMIS-MH scores, respectively (Figures 2, 3) [5,7]. A summary of the study outcomes is presented in Table 8.

**Table 8:** Summary of study outcomes BRS: Brief Resilience Scale; CD-RISC: Connor-Davidson Resilience Scale; PROMIS-PH: Patient-Reported Outcomes Measurement Information System-Physical Health; PROMIS-MH: Patient-Reported Outcomes Measurement Information System-Mental Health; KOOS JR: Knee Injury and Osteoarthritis Outcome Score Joint Replacement; EQ-VAS: EuroQol Group Visual Analogue Scale; EQ-5D: EuroQol Group 5 Dimension; WOMAC: Western Ontario and McMaster Universities Osteoarthritis Index

Author and year	BRS	CD-RISC	EQ scores	Osteoarthritis outcome scores	Length of stay	PROMIS-MH	PROMIS-PH	Patient-specific knowledge	UCLA	WOMAC
Bumberger et al. (2021) [11]	NR	72.9/100 (mean, pre-op)	NR	NR	NR	NR	NR	3.5/7 (mean, pre-op)	5.5 (mean, pre-op)	23.8 (mean, pre-op)
Bumberger et al. (2022) [12]	NR	69.5/100 (mean, post-op)	NR	NR	NR	NR	NR	3.8/7 (mean, post-op)	5.1 (mean, post-op)	2-months:22.3, 6-months: 18.4, 12-months:13.0 (all mean, post-op)
Haffar et al. (2021) [13]	4.17 (median, post-op)	NR	NR	50.0 (pre-op), 79.9 (post-op) (all median, KOOS-JR)	NR	NR	NR	NR	NR	NR
Lynskey et al. (2021) [14]	NR	34 (mean, post-op)	85 (median, post-op, EQ-VAS)	NR	NR	NR	NR	NR	NR	NR
Magaldi et al. (2019) [15]	3.73 (poor outcome group, pre-op), 3.54 (poor outcome group, post-op), 3.88 (high outcome group, pre-op), 3.93 (high outcome group, post-op) (all mean)	NR	0.688 (pre-op), 3-months: 0.750 (post-op), 12-months: 0.749 ( post-op) (all median, EQ-5D)	52.5 (pre-op), 3-months: 70.7(post-op), 12-months: 79.9 (post-op) (all median, KOOS-JR)	NR	53.3 (pre-op), 3-months: 53.3(post-op), 12-months: 50.8 (post-op) (all median)	44.9 (pre-op), 3-months: 50.8(post-op), 12-months: 50.8 (post-op) (all median)	NR	NR	NR
March et al. (2022) [16]	3.5 (mean, pre-op)	NR	NR	NR	6 (psychological symptoms group), 5 (psychologically well group) (all median)	NR	NR	NR	NR	NR
Nwanko et al. (2021) [17]	4.0 (pre-op), 4.0 (3-month post-op) (all median)	NR	NR	47.5 (pre-op) 66.0 (3-month post-op) (all median, KOOS-IS)	NR	50.8 (pre-op) 50.8 (3-month post-op) (all median)	39.8 (pre-op) 47.7 (3-month post-op) (all median)	NR	NR	NR
Trinh et al. (2021) [18]	4.0 (median, pre-op)	NR	NR	NR	NR	43.5 (low resilience, pre-op), 48.3 (normal resilience, pre-op), 53.3 (high resilience, pre-op); 45.8 (low resilience, post-op), 50.8 (normal resilience, post-op), 53.3 (high resilience, post-op) (all median)	37.4 (low resilience, pre-op), 39.8 (normal resilience, pre-op), 44.9 (high resilience, pre-op); 42.3 (low resilience, post-op), 47.7 (normal resilience, post-op), 54.1 (high resilience, post-op) (all median)	NR	NR	NR
Zabat et al. (2022) [19]	1-2.99 (low), 3-4.30 (normal), 4.31-5 (high)	NR	NR	72.5 (low resilience), 75.9 (normal resilience, 81.0 (high resilience) (all mean, HOOS-JR)	53.27 (low resilience), 38.70 (normal resilience, 25.64 (high resilience) (all mean)	NR	NR	NR	NR	NR

Discussion

This study aims to demonstrate that patients who have higher average levels of resilience are more likely to achieve better post-surgical outcomes following TJA. As previously discussed, psychological factors are known to play a significant role in both surgical and non-surgical health outcomes. In fact, the effect of resilience on improving outcomes within the field of orthopedics is not a novel phenomenon [19,20]. However, the specific role of resilience in maximizing the overall effectiveness of TJAs has not been extensively studied. This review seeks to address this gap by systematically reviewing existing data from nine studies that discuss both psychological resilience and post-surgical PROMs in TJA patients.

As expected, patients with higher baseline levels of resilience had enhanced short-term and long-term PROMs following the operation. These outcomes at three months post-surgery consistently showed that higher preoperative resilience, as measured by scales such as BRS and CD-RISC, correlated with improvements across various measures, including PROMIS-PH, PROMIS-MH, EQ VAS, WOMAC, and KOOS-JR. Notably, Trinh et al. and Lynskey et al. demonstrated significant positive correlations between resilience and physical and mental health outcomes post-TJA [18,7]. Longer-term outcomes, including those at six and 12 months, further supported the positive impact of preoperative resilience on postoperative recovery and function, with significant improvements reported in WOMAC, UCLA, and KOOS-JR scores. Additionally, the relationship between psychological factors and LOS was highlighted, with studies indicating that higher preoperative psychological stress, anxiety, and depression levels often corresponded to longer LOS. Conversely, higher resilience was linked to shorter LOS, especially in same-day discharge programs. Lastly, our meta-analysis validated that preoperative BRS scores and the various basal and postoperative patient-reported outcomes (PROMIS-PH, PROMIS-MH, KOOS) consistently had a strong positive correlation.

The findings from this systematic review closely align with existing literature on resilience in TJA. Numerous studies have consistently demonstrated that psychological preparedness and resilience are crucial factors in enhancing recovery outcomes following TJA. Our data suggest that in the context of TJA, resilient patients are better equipped to handle the physical and emotional demands of the surgery and recovery. They are more likely to adhere to rehabilitation protocols, maintain a positive outlook, and effectively manage pain and discomfort [21,22]. Ultimately, this review reaffirms the importance of psychological preparedness and resilience in enhancing recovery following TJA.

One considerable limitation of our review involves the variation in the measurement of resilience, as well as with PROMs. While most studies measured resilience using the Brief Resilience Scale (BRS), three of our included studies utilized the Connor Davidson Resilience Scale (CD-RISC). Moreover, there were over ten distinct scales that were used to measure PROMs. There was also notable clinical and methodological heterogeneity in patient populations and follow-up durations. Although statistical heterogeneity was assessed using I² and τ², and a random-effects model was applied, these differences may limit the comparability of pooled estimates. In addition, while no studies were deemed high risk of bias, the majority were rated as moderate risk using the MINORS criteria, which may introduce potential selection bias, confounding, and limitations in methodological rigor. Additionally, the overall certainty of evidence, as assessed using the GRADE framework, was low to very low across outcomes, further limiting the strength and generalizability of the conclusions.

A final limitation is the potential confounding from overlapping psychosocial constructs, such as depression, anxiety, and pain catastrophizing, which are known to influence postoperative outcomes and may correlate with resilience. Several included studies adjusted for these variables in multivariable analyses, while others reported unadjusted associations, introducing variability in the extent to which resilience represents an independent predictor of outcomes. As a result, the observed associations between preoperative resilience and postoperative PROMs may, in part, reflect the influence of these related psychosocial factors. Further, high-quality, prospective studies that aim to more consistently control for psychosocial variables are needed to better isolate the independent effect of resilience and strengthen the evidence base.

As all included studies were observational in design, the findings of this review support an association between preoperative resilience and postoperative outcomes rather than a causal relationship; therefore, conclusions regarding the direct impact of resilience on recovery should be interpreted with caution. Despite this, the findings of this review suggest that preoperative psychological resilience may be an important factor influencing recovery following total joint arthroplasty. Incorporating assessment of resilience into preoperative evaluation may help identify patients at risk for poorer postoperative outcomes. Additionally, targeted interventions aimed at improving resilience, such as prehabilitation programs or behavioral support strategies, may represent a potential approach to optimize recovery and enhance patient-reported outcomes.

Future research should focus on large, prospective studies to better define the independent effect of resilience on postoperative outcomes while controlling for confounding psychosocial variables such as depression, anxiety, and pain catastrophizing. Standardization of resilience measurement tools and patient-reported outcome measures is also needed to improve comparability across studies. Furthermore, interventional studies evaluating whether resilience-enhancing strategies can improve surgical outcomes would provide valuable insight into the potential clinical utility of targeting resilience in patients undergoing TJA.

## Conclusions

This review highlights the significant association between preoperative resilience and postoperative outcomes in patients undergoing TJA. The evidence consistently indicates that higher levels of resilience are associated with more favorable patient-reported outcomes, including reduced pain, improved physical function, and enhanced quality of life. Importantly, resilience is not simply an innate trait, but rather a quality that may be developed and strengthened through dedicated effort or, when appropriate, with support from a formally trained team and a caring physician. Thus, these findings underscore the potential value of assessing resilience before surgery. While incorporating resilience-building strategies into preoperative care may be beneficial, further prospective and interventional studies are needed to determine whether enhancing resilience directly improves recovery trajectories and patient satisfaction. Future research should continue to explore the mechanisms underlying these associations and evaluate interventions aimed at optimizing preoperative resilience.
